# Associated Effort

**Published:** 1874-03

**Authors:** N. W. Williams


### ASSOCIATED EFFORT.

BY N. W. WILLIAMS.

*Read before the American Dental Society of Europe.*

The Committee, in selecting the subjects for discussion, thought this the proper one for our first consideration in this the infancy of our Society.

The best argument in favor of associations in dentistry is, the standing of our profession in America, which owes its proud position, mainly, to its very many excellent societies. Every state in the Union has its State Association, which meets annually or semi-annually, besides many local societies. The Mississippi Valley Association, the oldest dental society in the world, which has had its annual sittings at Cincinnati for twenty-nine years, is composed of the prominent practical and solid men of the western and southern states. It has wielded an influence that has been felt throughout that section of country, and has placed the profession there in a position second to none in the Union. Then we have the National Association and, more recently, a Southern Branch, which have done and are doing much toward elevating the standard. There are nearly 100 Associations in the United States, New York having 17 and Ohio 9.

When we look at the men composing these associations, we find them to be the best operators and those who are steadily advancing toward perfection in our chosen art; while those who stand aloof, and feel so wise in their own conceit that it is impossible for them ever to learn anything more, are found to be so far in the rear as to be shocked at the rubber dam, and horrified at the mallet and burring engine, and to tell patients that they have had their teeth ground and pounded to death.

We are social beings; our Creator intended us to be such. What a dull existence this would be if we were all so selfish as to avoid contact with our fellow-men and, hermit-like, shut out the world and all its enjoyments! In our profession especially is it profitable to come together often to discuss points of practice and to give experiences. As an art, dentistry is so full of variety, nearly every day presenting cases with new and interesting phases, that we have more to tell—more to ask our brethren, each time we meet, and even one not at all in love with his profession can scarcely help feeling interested. Especially do we need the aid of associated efforts in this country, where our profession is only beginning to be felt and appreciated. We have much ignorance to combat and much prejudice to overcome, and those of us who have the elevation of the standard at heart should stand shoulder to shoulder and do all in our power to bring up the profession to that point to which our Societies in America have brought it.

### PREPARATION OF CAVITIES.

In the early days of our profession, our text-books taught and our preceptors advised, in the preparation of cavities, to remove as little of the tooth-structure as possible ; to simply scratch out the decayed matter, leaving the overhanging dentine and enamel ; so that, when the cavity was filled, there could be no chance of the filling coming out, especially if it was amalgam.

In the time of soft foil, many good fillings were put in, according to this method of preparing cavities, by careful and honest operators ; but it took much hard crowding : the tooth was made to support the filling, and as soon as the support was weakened by mastication or decay, the filling gave way.

When adhesive foil came into use, this mode of preparation was still more disastrous, as it was not possible to fill under projecting dentine or enamel so perfectly with adhesive as with soft foil, because there could not be so perfect an adaptation to the walls of the cavity : the consequence has been, that many teeth have been lost from insufficient cutting away of the projections ; by sparing the defective and sometimes sound dentine, a defective filling has been made.

In this enlightened age of dental science, I believe it is conceded by all good operators that no cavity is in proper condition to be filled unless there is free access to every part of the walls, so that a solid gold plug may be built from any given point to completion, leaving no chance for any part of the filling to stand away from the walls. The filling is made to support the remaining portion of the tooth, not the tooth to support the filling, as was the plan but a few months since.

But let me explain what I mean by "cut out the grooves:" A few years ago it was my good fortune to have in my possession for some weeks the fine and powerful microscope belonging to the Mississippi Valley Association; and, among other experiments, I placed a section of a perfect bicuspid under one of the objectives, and, to my great surprise, the little groove in the crown, hardly discoverable by the unassisted eye, presented a great fissure, capable of being the sink of all iniquities. Since then, I never feel satisfied to let a patient out of my hands, until I am fully convinced that the smallest drill will not penetrate the groove. I believe statistics would show that a larger number of bicuspids are lost than of the others, for which the profession is more responsible than the patient. The form of the tooth is such that it is sooner destroyed by decay than the others, and its position gives it more to do in masticating the food, as well as in rending and biting it. Often upon examination these teeth are passed by, if only a dark line is discovered, and the patient is told that that tooth will require attention in six months or a year; but, as the patient rarely returns as soon as advised, often both he and the operator are surprised to find the poor little bicuspid a hopeless wreck: while if it had been filled as soon as the smallest and sharpest drill could penetrate the little groove, this lamentable result would have been prevented, and, as is often the case, the discovery might have been made that the two or three little pits, when drilled into, are not only deep and on the way to the nerve, but frequently communicate with approximal cavities that had not been discovered before.

To make a good filling in a tooth decayed in this manner, the groove on the crown must be cut out to a well-defined shape and made to communicate with the approximal cavities; make a good foundation at the point near the gum to begin the plug: if properly filled, the little bicuspid has a chance of a long and useful career; but if filled as it usually is, by drilling out each little pit and making separate fillings, in the majority of cases the result is, not success, but failure.

How often do we see the molars having a little, pin-head gold filling showing in the center, with dark lines running from it, like points from a star, which on examination are found softened by decay, and the gold plug entirely undermined. In nearly all molars we find these dark lines radiating from the central filling, more or less deep; if these are not cut out when preparing, and the two, three or four pits or grooves made into one cavity and all filled with one solid plug, sooner or later that filling will be a failure. In the days gone by, before the burring engines were invented, to cut out the grooves was a difficult task, and gave some excuse for leaving them until decay had made it easier; but now there is no excuse, for with chisel and burring engine we can shape them to suit the most fastidious operator, and make the teeth serviceable for life, while by the old way their loss was only a question of time.

